# Ultrathin Assembles of Porous Array for Enhanced H_2_ Evolution

**DOI:** 10.1038/s41598-020-59325-4

**Published:** 2020-02-11

**Authors:** Aminul Islam, Siow Hwa Teo, Md. Rabiul Awual, Yun Hin Taufiq-Yap

**Affiliations:** 1 Chancellery Office, Universiti Malaysia Sabah, 88400 Kota Kinabalu, Sabah Malaysia; 2 Department of Petroleum and Mining Engineering, Jashore University of Science and Technology, Jashore, 7408 Bangladesh; 3 Catalysis Science and Technology Research Centre, Faculty of Science, Universiti Putra Malaysia, 43400 UPM Serdang, Selangor Malaysia; 4 Materials Science and Research Center, Japan Atomic Energy Agency (JAEA), Hyogo, 679–5148 Japan

**Keywords:** Energy science and technology, Materials science

## Abstract

Since the complexity of photocatalyst synthesis process and high cost of noble cocatalyst leftovers a major hurdle to producing hydrogen (H_2_) from water, a noble metal-free Ni-Si/MgO photocatalyst was realized for the first time to generate H_2_ effectively under illumination with visible light. The catalyst was produced by means of simple one-pot solid reaction using self-designed metal reactor. The physiochemical properties of photocatalyst were identified by XRD, FESEM, HRTEM, EDX, UV-visible, XPS, GC and PL. The photocatalytic activities of Ni-Si/MgO photocatalyst at different nickel concentrations were evaluated without adjusting pH, applied voltage, sacrificial agent or electron donor. The ultrathin-nanosheet with hierarchically porous structure of catalyst was found to exhibit higher photocatalytic H_2_ production than hexagonal nanorods structured catalyst, which suggests that the randomly branched nanosheets are more active surface to increase the light-harvesting efficiency due to its short electron diffusion path. The catalyst exhibited remarkable performance reaching up to 714 µmolh^−1^ which is higher among the predominant semiconductor catalyst. The results demonstrated that the photocatalytic reaction irradiated under visible light illumination through the production of hydrogen and hydroxyl radicals on metals. The outcome indicates an important step forward one-pot facile approach to prepare noble ultrathin photocatalyst for hydrogen production from water.

## Introduction

Why do we need to promote hydrogen energy? The scientist lays out about four main reasons-energy-saving, minimal ecological impacts, energy security and industrial competitiveness. Industrially, fossil-fuel based hydrogen production process emits greenhouse gas to the environment^1^. The use of solar energy to produce hydrogen from water could accelerate the development of high-impact breakthrough clean energy technologies. By developing an optimal photocatalyst, scientists are searching for the ways of improving clean energy (H_2_) production from water without producing greenhouse gases or having many adverse effects on the atmosphere.

A variety of titanium dioxide (TiO_2_) phases and nanostructures have been studied extensively for photocatalytic hydrogen production because of its earth-abundance, non-toxicity as well as thermal and chemical stability^1,2^. However, photoirradiated electron recombination and wide band gap of bare TiO_2_ remain challenge on efficient hydrogen production. Much work has been directed towards the adaptation of noble Pd, Au metals on the TiO_2_ metals^3–5^ or through the use of sacrificial reagents^6^. Noble co-catalyst could serve as electron sinks to isolate the photogenerated electron-holes^3^. Moreover, noble metals assist to boost the reaction process by lowering over-potential for proton deduction^4^. Unfortunately, the state-of-the-art co-catalysts are still noble metals (e.g. Pt, Au, Pd) or their oxides that are rare and expensive. As a consequence, the discovery of robust, low-cost and earth-abundant co-catalysts as substitutes of noble metals remains a great challenge for photocatalytic hydrogen production.

A layered molybdenum disulfide (MoS_2_) has been reported for H_2_ evolution through photoirradiated water splitting^7,8^. However, the scarcity and complex with time-consuming fabrication steps of catalyst void their competitiveness and hamper their widespread use in industry. Therefore, fabricating of simple and reliable procedures for efficient water splitting is still desirable. Recently, attention has been paid to progress towards ferroelectrics material for hydrogen evolution under solar light illumination because of its nontoxicity and low cost such as Fe/Ni/BaTiO_3_^9^, HfOx/SiOx^10^ and WO3/MIL-101^11^. However, the hydrogen production efficiency of ferroelectrics based photocatalyst is still low due to poor electrical conductivity. Many efforts have been focused to develop nitride complex (g-C_3_N_4_) supported catalyst for H_2_ generation through water splitting under illumination with visible light^12–15^ Layered graphitic g-C_3_N_4_ catalyst was demonstrated by Botari *et al*.^16^ for efficient photocatalytic H_2_ production from water. Che *et al*.^17^ have elaborated an effective cocatalyst of graphitic carbon ring for g-C_3_N_4_ owning to its layered structure that facilitates the separation of photoexcited electron–hole efficiently. However, the efficiency can be increased by applying PtH_2_PtCl_6_ solution and thus the wild use of noble catalyst is restricted by its high cost. In addition, the C_3_N_4_ catalyst suffers from poisoning by the produced H_2_O_2_ during water splitting^18^.

The groundbreaking research in developing photocatalyst is the synthesis of highly effective catalyst from earth abundant materials for the H_2_ production from water. Efficient earth-abundant nickel-based chalcogenides attracted attention for H_2_ generation through water splitting^19^. Pioneering works by Indra *et al*.^20^ and Luo *et al*.^21^ have reported the semiconductors supported molecular nickel complexes for efficient photocatalytic hydrogen production. In addition, CdS/Ni_x_S_y_^22^, Ni-hexacyanoferrate^23^, Ni/SiC^24^ and Ni/SrTiO_3_^25^ systems with semiconductors have also been reported with moderate activity. To date, although many noble metal-free cocatalysts have been focused capable of producing hydrogen from water without adjusting pH, applied voltage, sacrificial agent or electron donor, but the efficiency is low enough to equate with the reported state of art noble photocatalyst.

Recently, synthesis of ultrathin (<10 nm) metallic assembles has attracted considerable interest due to their unique photocatalytic properties^26^. In particular, the intensive research attention has been paid to fabricate interconnected open-pore structured nanosheet owing to their maximum exposure of the active sites and fast mass/electron transfer ability^27^. The 2D Ru nanosheet and their 2D oxide derivative found to be robust catalysts for photocatalytic water splitting reaction^28^. Vertically aligned WS_2_ nanosheet with open and porous framework exhibited significantly improved catalytic performance in the water splitting compared with the commercial nanostructured WS_2_^29^. Photochemically reduced Pt deposited on TiO_2_ nanosheet was showed efficient photocatalytic activity under irradiation with xenon lamp^30^. Many noble metal based nanosheet photocatalysts were also reported to be efficient towards the water splitting, e.g. Pt/CoP^27^, CdS nanosheet^31^, Pt/TiO_2_^32^, Au/MoS_2_^33^, RuOx/Ca_2_Nb_3_O_10_^34^ and so on. Predominantly, noble metals (Pt, Ru, Ge, Se) modified nanosheet photocatalysts were used extensively for efficient water splitting reactions. However, the shortage and significant expense of precious metals limit their functional application in enormous scale. Additionally, the synthesis of semiconductor based nanosheet assembles is very complex owing to the electrostatic attraction force between adjoining nanosheet^35^. Although many progresses have been achieved, it is still highly desirable to develop facile strategies for controllable and well-defined semiconductor based nanosheet assemblages.

Attempt has been made in this work to develop effective ultrathin structured porous photocatalyst from earth abundant metals. A noble ultrathin assembles of Ni-Si/MgO photocatalyst synthesized by a facile one-pot solid phase reduction method which could reduce the complexity of fabrication. As far as we could possibly know, there is no report on investigating efficient and low-cost nanosheet assemblages for water splitting. The catalyst was illuminated under visible light irradiation avoiding any sacrificial reagent. The effect of structure and surface function on photocatalytic efficiency was discussed in this study. The catalyst exhibited remarkable performance which is higher than the prevailing semiconductor catalyst.

## Results and Discussion

**Figure 1:**
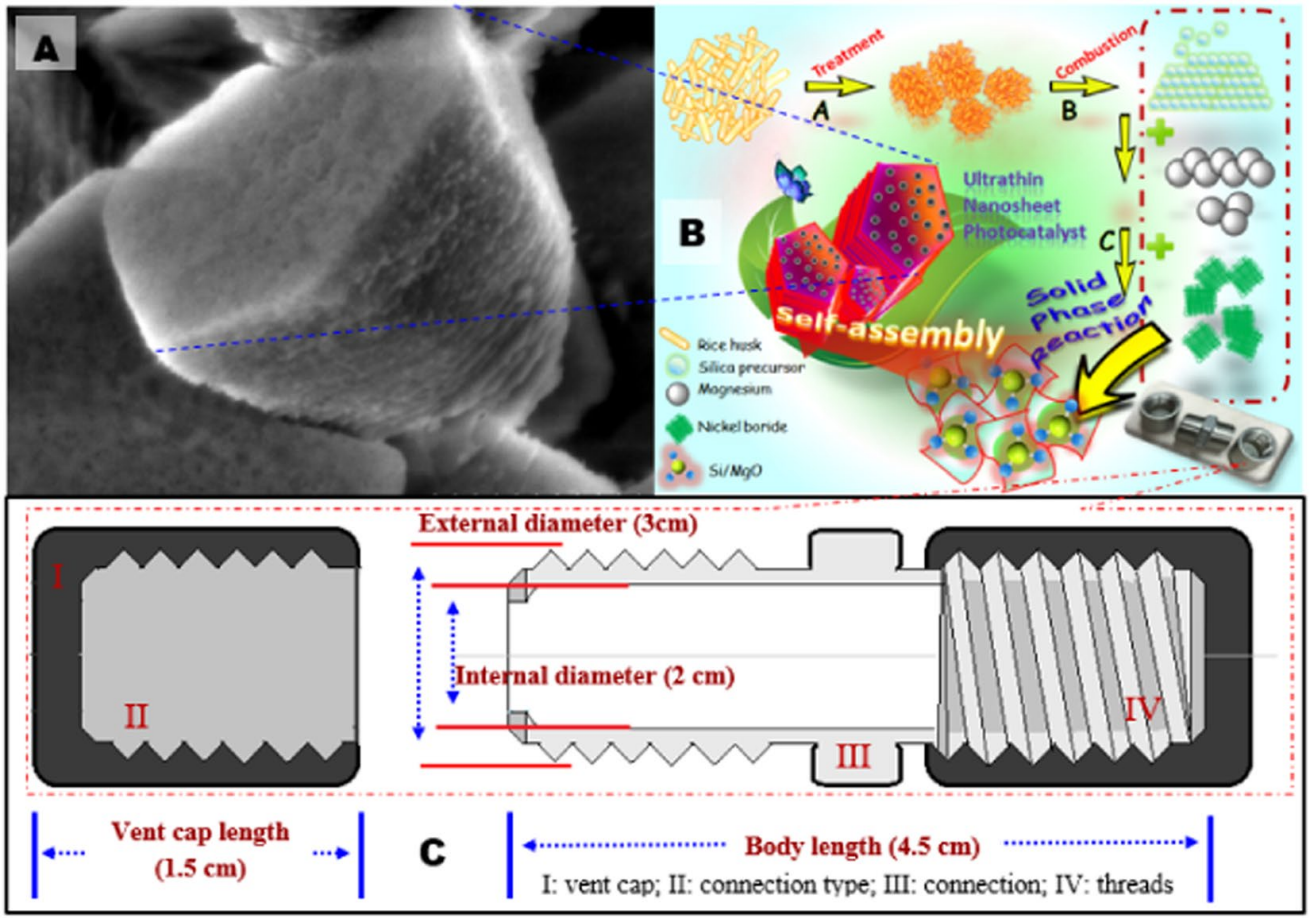
(**A**) Illustration of the preparation of the Si/MgO by the solid phase reaction method; (**A**) Treatment of rice husk; (**B**) conversion of silica from rice husk; (**C**) photocatalyst was prepared using solid phase reaction in which SiO_2_ and NiB were used as a precursor. (**b**) Photo of the homemade reactor design used to perform solid phase reaction to produce photocatalyst.

Silica powder was obtained by wet acid and thermal treatment at 800 °C for 3 h. The powder was subjected to the reaction with hydrochloric acid and hydrofluoric acid to generate hydrogen bond onto the metal surface^36^. Electron-deficient hydrogen bond on metal surface could act as electron sink, leading to improve charge detachment^37^. Ni was prepared by solvothermally treating sodium borohydride and nickel chlorite in ice water bath at 5–7 °C for 3 h. MgO supported Ni-Si photocatalyst was prepared by one-pot solid phase reduction process at 750 °C for 3 h to prepare nanosheet assembled porous structure (Fig. 1A,B). MgO support modifies not only the electronic state of the active sites and the chemisorption ability of surface functional group due to its acid-base character, but also the morphology of the metal crystallites^38^. In addition, the active metal components disperse highly onto support material which is directly related to the catalytic activity^37,38^. Hence, MgO supported Ni-Si nanosheet assemblages was synthesized by reduction method using a stainless steel reactor capped with two ends, as depicted in Fig. 1C.

**Figure 2:**
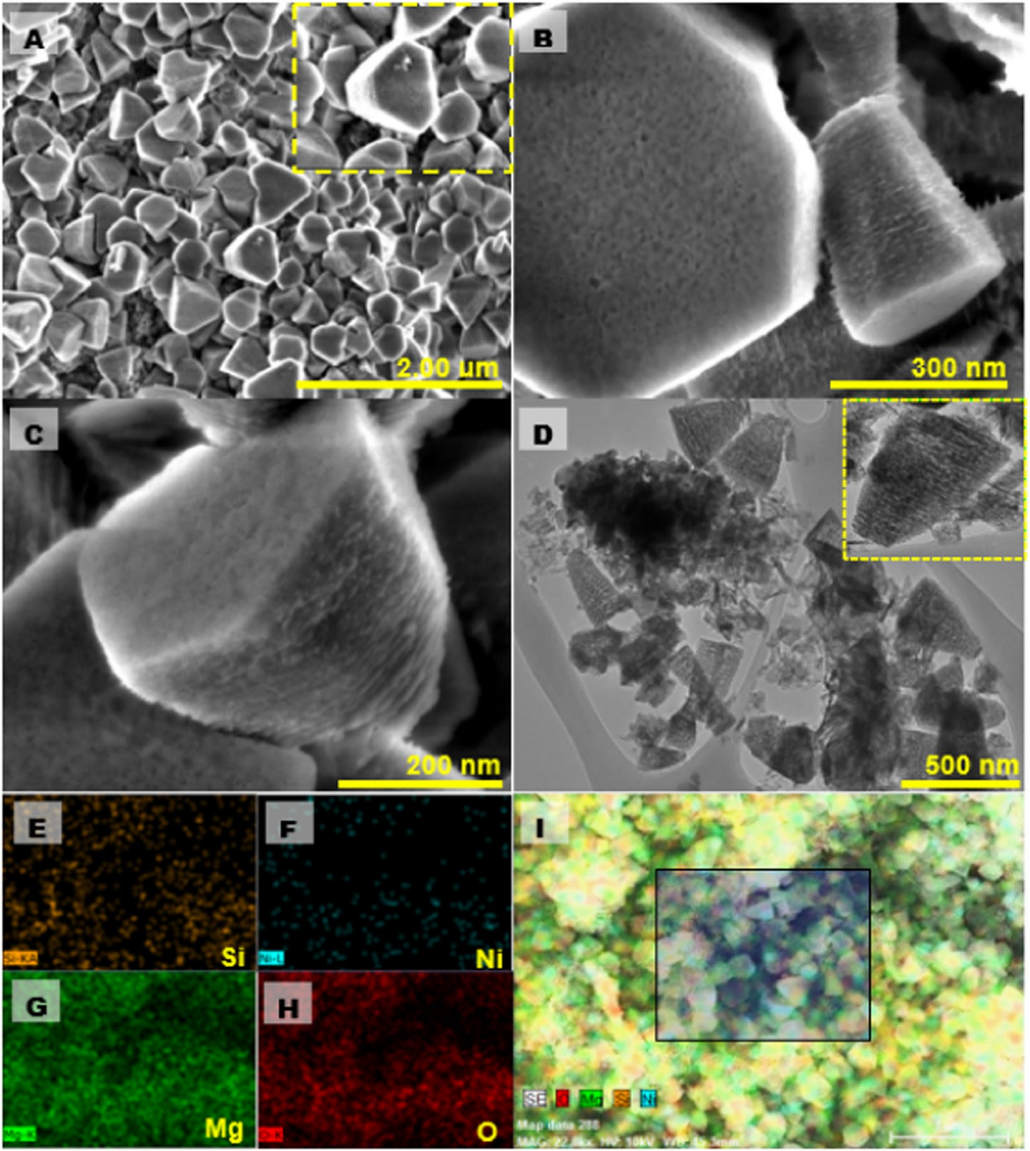
Morphological characterization of ultrathin 0.5Ni-Si/MgO nanosheets. (**A–C**) Scanning electron micrograph of 0.5Ni-Si/MgO catalyst. Inset: magnified SEM image of catalyst. (**D**)Transmission electron micrograph of 0.5Ni-Si/MgO catalyst. Inset: magnified TEM image of nanosheet catalyst. (**E–I**) SEM-EDX elemental mapping images showing Si distribution (**E**), Ni distribution (**F**), Mg distribution (**G**) and O distribution (**H**) and SEM.

Morphological evaluation of the synthesized catalyst was performed by FESEM and HRTEM. It can be seen (Fig. 2A) that as prepared 0.5Ni-Si/MgO sample consists of three-dimensionally vertical inter grown stacking of ultrathin-nanosheet. Notably, the orderly hexagonal nanosheet arrays with much increased coverage of porous structure were observed (Fig. 2B,C). The distribution of porous channels in the entire nanosheets may be associated with the improved transport rate of photocarriers^39,40^.

**Figure 3:**
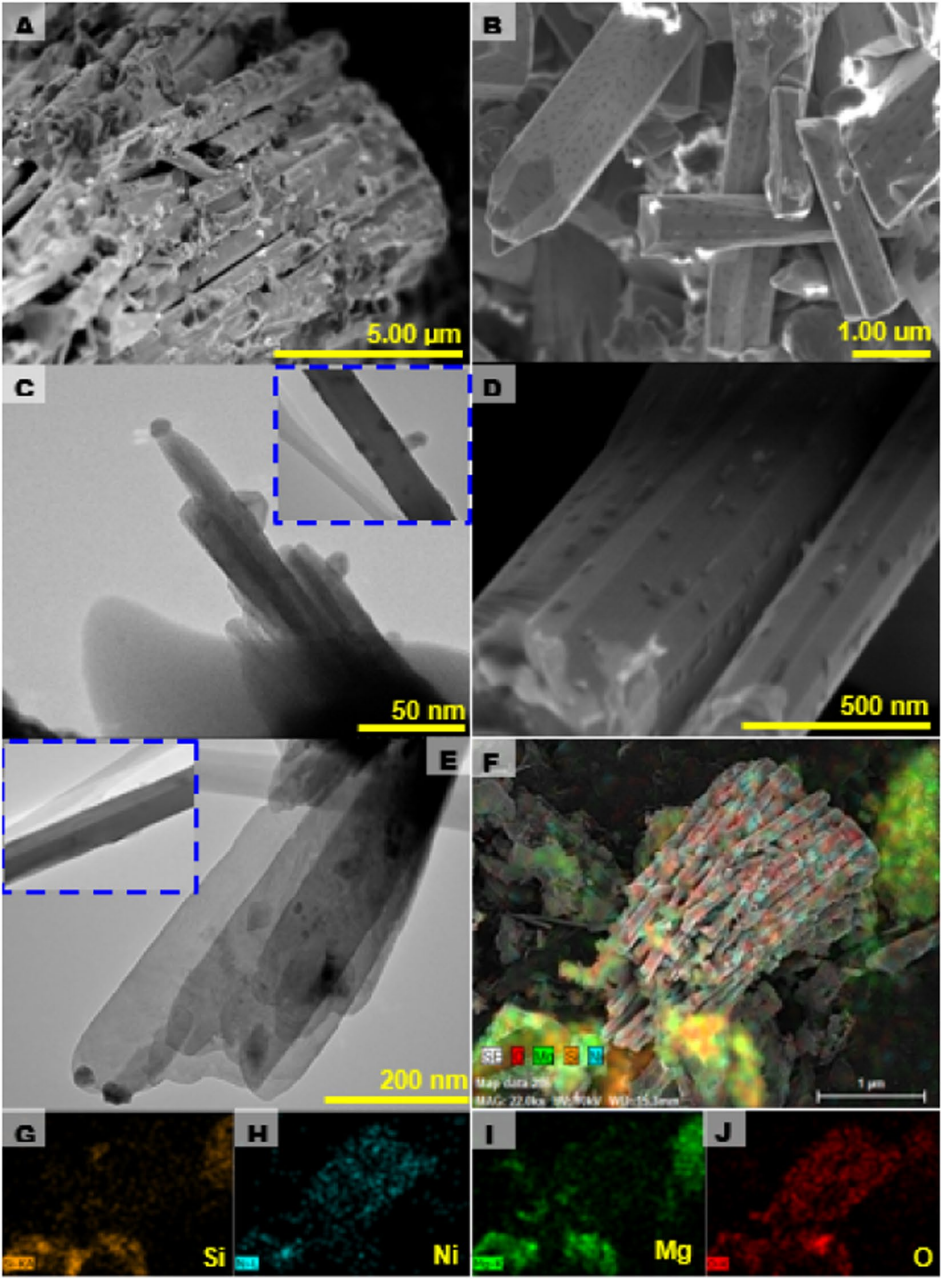
Morphological characterizations of 0.7Ni-Si/MgO nanorods. (**A–C**) Scanning electron micrograph of 0.7Ni-Si/MgO catalyst. Inset: magnified SEM image of catalyst. (**D**,**E**)Transmission electron micrograph of 0.7Ni-Si/MgO catalyst. Inset: magnified TEM image of nanorods catalyst. (**F–J**) SEM-EDX elemental mapping images showing an SEM image (**F**), Si distribution (**G**), Ni distribution (**H**), Mg distribution (**I**) and O distribution (**J**).

Figure 2D shows the HR-TEM image of ultrathin-nanosheet structure which is well consistent with FESEM results. The self-assembled nanosheet geometry could be formed by the interaction of conjugated molecules in a planar geometry operated by the van der Walls forces^41^. EDX mapping of 0.5Ni-Si/MgO catalyst demonstrated the presence of O, Mg, Si and Ni elements (Fig. 2E–H). The uniform distribution of Ni and Si on MgO support in nanosheet structures was further confirmed by EDX mapping, as shown in Fig. 2I. The uniform dispersion of co-catalyst in the layered structure which can be explained by the fact that, more catalytically active sites were formed on the sample surfaces^42^. With increasing Ni concentration from 0.5 to 0.7 wt%, the morphology of 0.7Ni-Si/MgO sample significantly changed to three-dimensional hierarchically rod-like bundle nanostructure, as observed in Fig. 3A. The approaches for stability of lamellar and rod structures have been discussed in terms of the mechanisms by Jackson and Hunt^43^. According to Jackson and Hunt^43^ the motion of lamellar spacing during the solid phase reaction causes the termination of lamellar structure. Since the termination absorbs any effective radius or any shape, in spite of the lamellar spacing, it perhaps produces rod shape upon cooling. Notably, the amplified region in Fig. 3B,C shows the formation of well-defined staking nanorods with hexagonal crystal structure. The hexagonal nanostructure assembled of nanorods with an average diameter of 30 nm, length of 110 nm was confirmed by HR-TEM image (Fig. 3D,E) and thus addressed the elemental mapping across the nanorods by means of EDS (Fig. 3F–J). The EDX spectrum in Fig. 3G–J manifests the coexistence of Si, Ni, Mg and O elements. The special distributions are depicted in the EDS mapping images (Fig. 3F) of the selected area. It suggests that the Si and Ni co-catalysts are uniformly distributed along the length of the nanorod. It is deserving of note that the development of nanorod with well-defined hexagonal structure was reported by other workers by using expensive patterning methods^44,45^.

**Figure 4:**
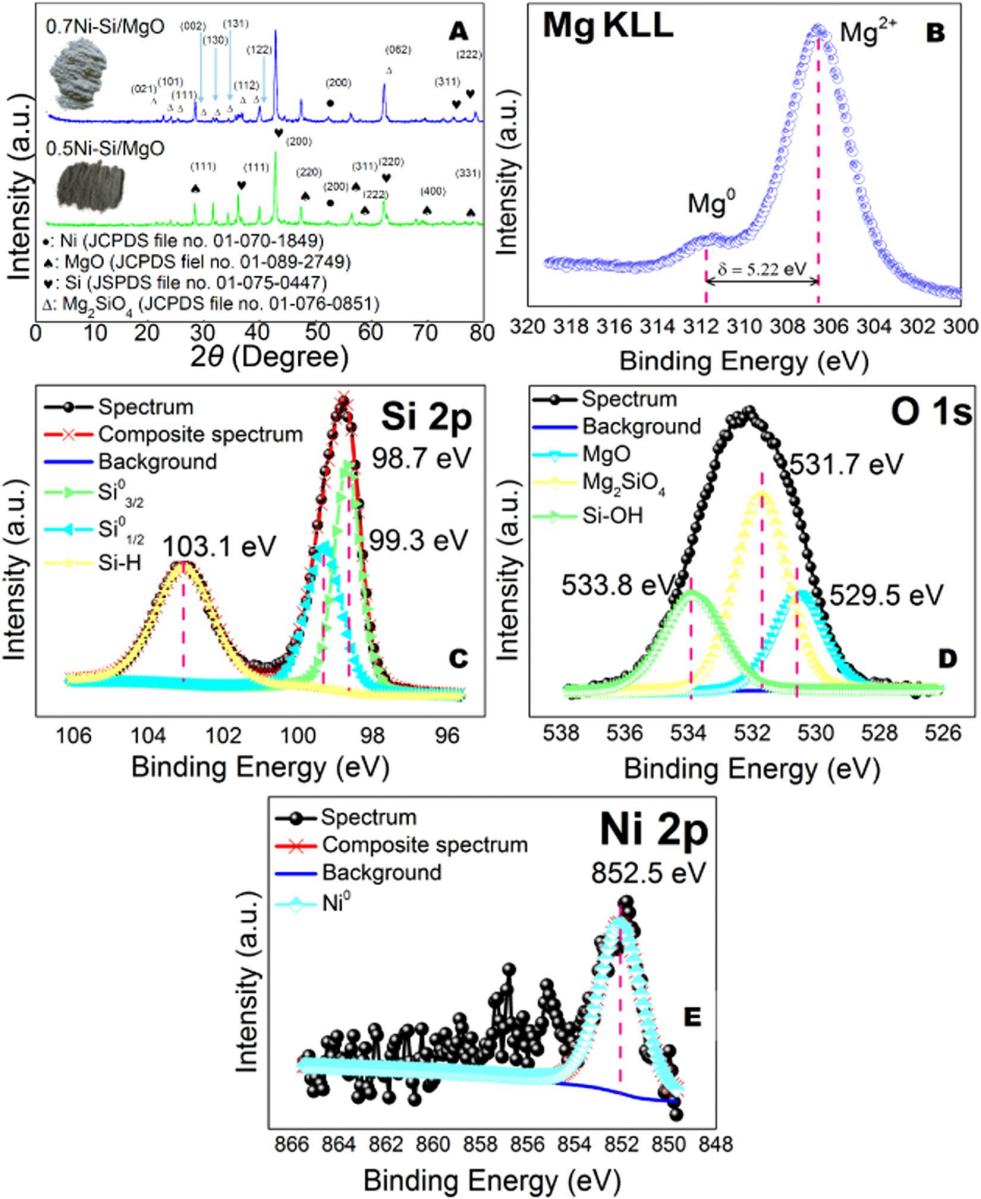
Structural and chemical characterization of catalyst. (**A**) X-diffraction of 0.5Ni-Si/MgO catalyst. (**B**) XPS spectra of Mg KLL (**C**) XPS spectra of Si 2p. (**D**) XPS spectra of O 1 S. and (**E**) XPS spectra of Ni 2p for 0.5Ni-Si/MgO catalyst.

X-Ray diffraction was conducted to gain information on the crystal structure of photocatalyst. As shown in Fig. 4A, the diffraction peak for 0.5Ni-Si/MgO catalyst at 28.56°, 47.37°, 56.54°, 58.02°, 69.38°, 77.05° attributed to the MgO as confirmed by JCPDS file no. 01-089-2749. The crystal planes of Si (JCPDS file no. 01-075-0447) was identified as the principal phase (2*θ* of 36.26° (111), 62.32° (220), 74.79° (311) and 78.64° (222)) for the sample showed in Fig. 4A. The weak diffraction peaks at the 2*θ* of 52.12° (200) in the spectra of sample match well with the Ni phase (JCPDS file no. 01-070-1849). The peaks of the Mg_2_SiO_4_ (2*θ* of 22.89°, 24.25°, 24.50°, 29.80°, 31.84°, 34.45°, 34.94°, 40.11°, and 62.78°, JCPDS file no. 01-076-0851) on catalyst show ten crystal planes, corresponding with 021, 101, 111, 002, 130, 131, 121, 122, and 062 facets. The resulting hierarchically ultra-thin 0.5Ni-Si/MgO nanosheets were then characterized by XPS. As shown in Fig. 4B, the Mg KLL XPS Auger peaks were formed after the magnesiothermic reduction process. The binding energy for metallic magnesium identified at 311.8 eV is shifted by about 5.22 eV towards higher binding energies after solid phase reaction associate with the formation of magnesium oxide^46^. The deconvolution of the Si2p peak (Fig. 4C) gives peaks at 98.7, 99.3 and 103.1 eV, which belong to Si^o^ and Si–H, respectively^47^. The presence of Si–H bonds on the catalyst surface is beneficial for enhancing the separation of photogenerated charge carriers in the nanosheets photocatalyst and act as hydrogen production sites^18^. The photogenerated electron can react quickly with the Si–H group from a semiconductor to form H_2_ and thus the transfer of holes in nanosheet of catalyst could be accelerated^18,48^.

As shown in Fig. 4D, the deconvoluted O1s peak at ∼533 eV corresponds to Si–OH bond implying the existence of Si–OH embedded in the nanosheet photocatalyst^18,48^. The unstable dangling H bonds could be associated with the formation of Si–OH due to surface oxidation^18,49,50^. The formation of Si-H and Si-OH bonds on the Si nanosheets is consistent with the recent reports^47^. The O1s satellite peak at 531.7 eV is due to Si^4+,^ indicating the presence of magnesium silicates species while the lower binding energy peak at 529.5 eV attribute to MgO shown in Fig. 4D^47,51,52^. The deconvoluted Ni2p peak at binding energies of 852.5 eV corresponds to Ni metal shown in Fig. 4E which agrees well with those reported for Ni metal^53^.

**Figure 5:**
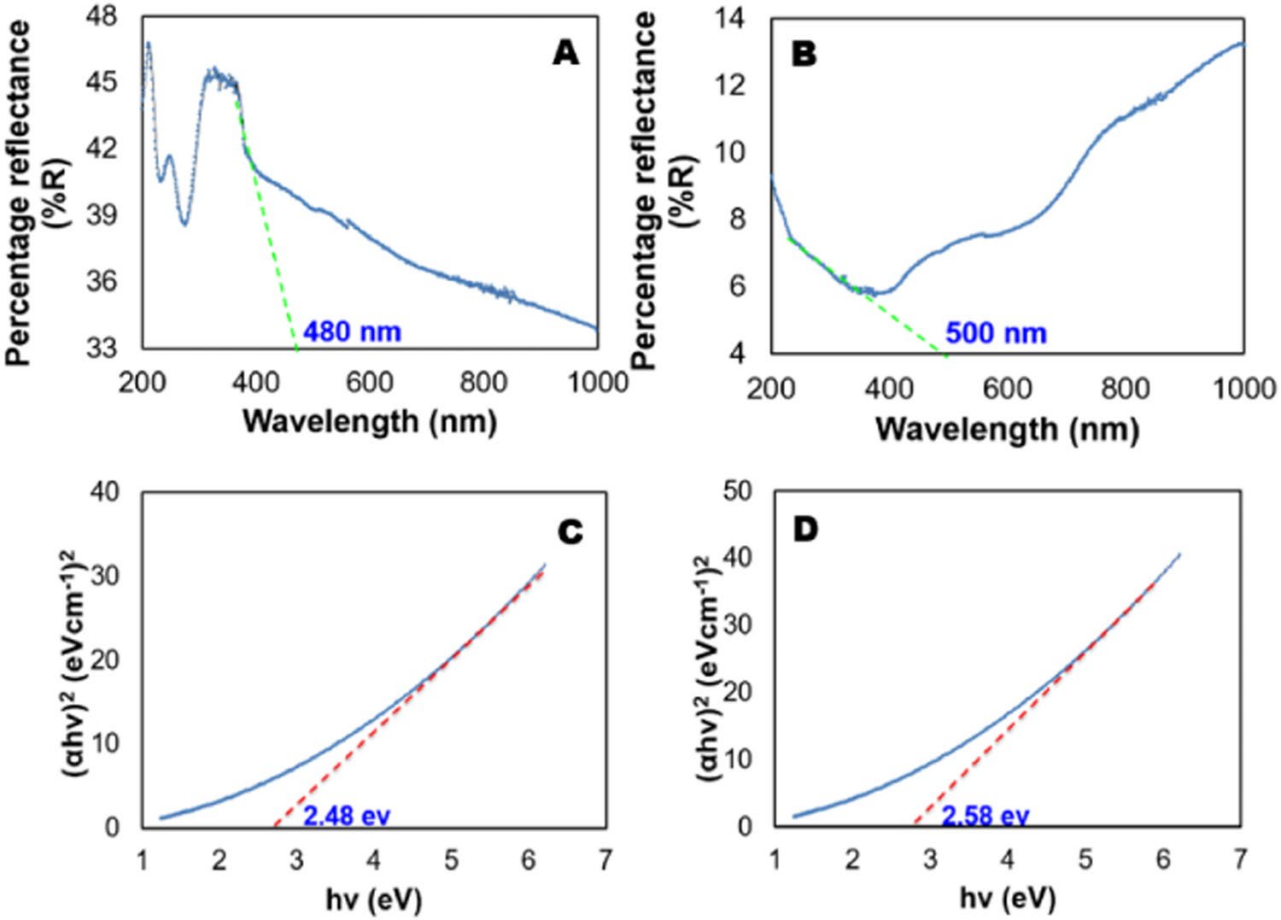
Optical absorption analysis of the photocatalyst: (**A**) 0.5Ni-Si/MgO catalyst, (**B**) 0.7Ni-Si/MgO catalyst and Tauc plots and band gap determination of (**C**) 0.5 Ni-Si/MgO catalyst, (**D**) 0.7 Ni-Si/MgO catalyst.

The bandgap of the photocatalyst was determined using direct semiconductor formula. Upon recognizing electron-energy-loss spectroscopic results, Goniakowski *et al*.^54^ concluded that the supported or unsupported MgO was found to exhibit semiconductive behavior. By using different semiconductors in appropriate proposition, Scagnelli *et al*.^55^ demonstrated that MgO supported catalyst showed a great flexibility for bandgap due to its semiconductive nature. More recently, Fuku *et al*.^56^ was used a direct semiconductor formula to determine the bandgap of NiO/MgO nanocomposite. Hence, the method for band gap determination used in this study is consistent with the experimental data compiled by other researchers^54–56^. The results of the absorption spectra of the catalyst was provided in Fig. 5A,B. The absorption edges of 0.5Ni-Si/MgO and 0.7Ni-Si/MgO catalyst were located at approximately 500 nm and 480 nm respectively which is in agreement with its characteristic absorption in visible spectrum range^57^. Here, the optical band gap energies of 0.5Ni-Si/MgO and 0.7Ni-Si/MgO were calculated from Tauc plots according to the relationship αhυ = C(hυ − E_g_)^2^ or (αhυ)^2^ = ${{\rm{C}}}^{\text{'}}$(hυ − E_g_), where α is the coefficient of absorption, hυ is the constant and E_g_ is the band gap energy^58^, respectively. The evaluation of absorption coefficient was determined by α = Kln(R_max_-R_min_/R-R_min_), where, maximum reflectance is the R_max_ and the minimum reflectance is R_min_^58^. The estimation of band gap was performed by the plot (αhυ)^2^ versus photon energy (hυ) and bandgap values for the 0.5Ni-Si/MgO and 0.7Ni-Si/MgO measured by the curve fitting were 2.48 eV and 2.58 eV respectively, as shown in Fig. 5C,D. The results demonstrated that the band gap of Ni-Si/MgO phocatalyst can be adjusted by tuning the cocatalyst concentration, which accordingly leads to their different efficiency in photocatalytic reactions.

The identified Si and Ni metals (M) of photocatalyst and their M-H and M–OH bonds on ultrathin layers of catalyst could be associated with the oxidation/reduction reaction during hydrogen production. The efficiency of semiconductors modified photocatalyst can greatly be affected by their states of surface radicals^47^. After simulation of adsorption and dissociation of water molecules on Si-H, Si-OH and Si surface, a conclusion has been reached by Zhang *et al*.^57^ that the dissociation of water would take place preferentially on Si surface when irradiate in water. The holes on metals surface (Si or Ni) (M-h^+^) could act as oxidation center (Eq. 1) where H^+^ would trap by Si-H bonds to provide proton reduction sites for H_2_ production^18^ (Eq. 2).

1 $${\bf{O}}{\bf{x}}{\bf{i}}{\bf{d}}{\bf{a}}{\bf{t}}{\bf{i}}{\bf{o}}{\bf{n}}:{{\rm{h}}}^{+}+{\rm{M}}+{{\rm{H}}}_{2}{\rm{O}}\to {\rm{M}}-{\rm{OH}}+{{\rm{H}}}^{+}$$

2 $${\bf{R}}{\bf{e}}{\bf{d}}{\bf{u}}{\bf{c}}{\bf{t}}{\bf{i}}{\bf{o}}{\bf{n}}:{{\rm{e}}}^{-}+{\rm{M}}-{\rm{H}}+{{\rm{H}}}^{+}\to {\rm{M}}+{{\rm{H}}}_{2}$$

Other workers^47^ have discussed a similar phenomenon and have suggested that the released H^+^ cations are supposed to pick up photoexcited electrons on the surface of M-OH and M-H for the reduction through the reaction (Eq. 2). Examining the mechanism of surface charge behavior and reaction mechanism of hydroxide on semiconductor metals, Liu *et al*.^18^ concluded that the formation of OH group onto the metal surface may prefer to form a new H_2_O molecule by consuming photoexcited electron as shown in Eq. 3, resulting in low probability of generating oxygen. It may be mentioned that the suppression of H_2_ production could be attributed to proceed by reaction (Eq. 3).

3 $${\bf{R}}{\bf{e}}{\bf{d}}{\bf{u}}{\bf{c}}{\bf{t}}{\bf{i}}{\bf{o}}{\bf{n}}:{{\rm{e}}}^{-}+{\rm{M}}-{\rm{OH}}+{{\rm{H}}}^{+}\to {\rm{M}}+{{\rm{H}}}_{2}{\rm{O}}$$

**Figure 6:**
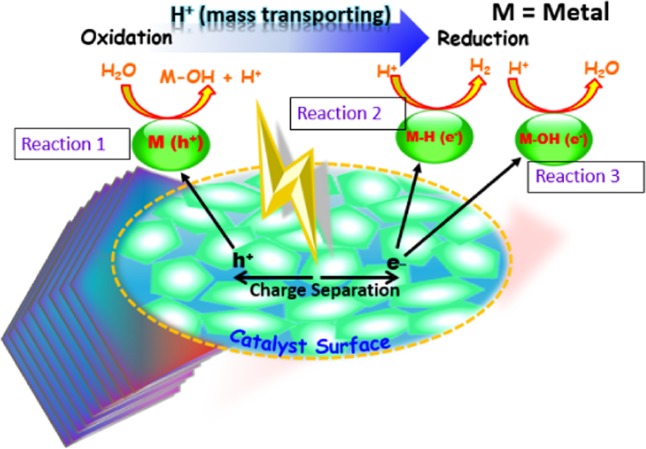
The oxidation/reduction reaction on the catalyst surface.

Likewise, the formation of OH group onto the Ni surface may prefer to form a new H_2_O molecule by hosting the reaction (Eq. 3), resulting in low probability of generating oxygen. The oxidation/reduction reaction on catalyst is shown in Fig. 6.

The catalytic reactions indeed produce only H_2_ gas from water in the present study as shown in Fig. S1. This observation is consistent with the results demonstrated previously by Liu and coworkers who reported silicon nanowires as the starting materials for photocatalytic water splitting^18^. Therefore, the results indicate that the production of H_2_ may not take place along a conventional water-splitting process. The data for H_2_ production agree quite well with the results reported by other researchers^18,58^.

**Figure 7:**
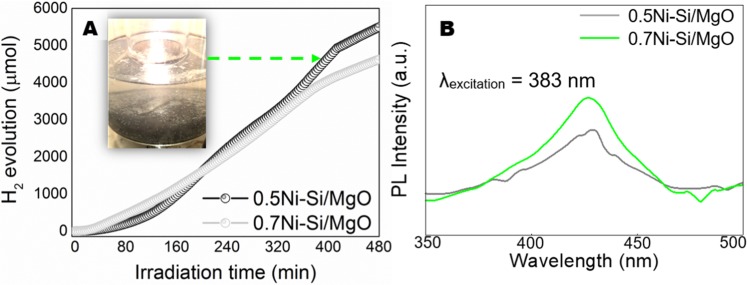
(**A**) Comparative photocatalytic activity of photocatalyst, 0.5Ni-Si/MgO and 0.7Ni-Si/MgO. The system was irradiated by visible light without using any sacrificial reagent. (**B**) PL spectra of the photocatalyst.

Figure 7A shows the time-dependent photacatalytic H_2_ generation through water splitting obtained from 0.5Ni-Si/MgO and 0.7Ni-Si/MgO samples. As shown in Fig. 7A, higher photocatalytic H_2_ evolution rate of 0.5Ni-Si/MgO (714 µmolh^−1^) was observed compared to the 0.7Ni-Si/MgO (611 μmolh^−1^). The structural and morphological characteristics of photocatalyst may attribute to compare the differences of catalytic efficiency. Evidently, the dominance of Si phase in the 0.5Ni-Si/MgO sample might be associated with the higher activity compared to Mg_2_SiO_4_ phase for 0.7Ni-Si/MgO sample (Fig. 4A). This is in agreement with the difference in photogenerated carriers of the reactive metal/metal-compound^59^. As shown in Fig. S2A–D, there was no observable change in the structure of 0.7-Ni-Si/MgO catalyst. However, the Ni(OH)_2_ phase noticed from the Fig. S2E could be ascribed to the lower activity of 0.7-Ni-Si/MgO catalyst. Examining the mechanism of surface charge behavior and reaction mechanism of hydroxide on semiconductor metals, Liu *et al*.^18^ concluded that the formation of OH group onto the metal surface may prefer to form a new H_2_O molecule by consuming photoexcited electron as shown in Eq. 3, resulting the suppression of H_2_ production rate. A similar observation was made by other researchers^60,61^ who studied extensively on the H_2_ generation over semiconductor photocatalyst.

In addition, the synthesised nanosheet assemble structures (Fig. 2) could be attributed to the higher photocatalytic activity than the nanorods (Fig. 3). Thus, the question may arise: why do ultrathin 0.5Ni-Si/MgO nanosheets exhibit a much higher activity than 0.7Ni-Si/MgO nanorods? Many studies have demonstrated that the photoexcited electrons and holes may be increased by multiple reflections of light in the nanosheet assemblages and thus enhancing the photocatalytic reactions^62^. The light-harvesting of nanosheets superstructure could be enhanced by multiple reflections of light and thus, increases the photoexcited electrons and holes to contribute the photocatalytic raections^62^. The lower photocatalytic H_2_ production in 0.7Ni-Si/MgO nanorods could be a consequence of poor intralayer charge transport. Further, the drastic enhancement of photocatalytic activity for ultra-thin nanosheets is attributed to the following factors:^17,63,64^ i) The holey 2D ultrathin structure of 0.5Ni-Si/MgO nanosheets can provide more exposed new catalytic active sites which maximizes the cross-plane diffusion of photogenerated carriers; ii) Short migration path of ultrathin nanosheets for photogenerated electron may results in high activity; iii) a dense network of crystalline nanosheets may decrease the electron diffusion length and provides enhanced photon absorption to the catalyst; (iv) the randomly branched nanosheets may increase light-harvesting efficiency by scattering enhancement and trapping. As is well-known, the photocatalytic activity is closely related to photoexcited electrons-hole generation. The 0.7Ni-Si/MgO nanorods exhibited a strong photoluminescence (PL) peak, suggesting the fast carrier recombination (Fig. 7B)^65^. In contrast, the lower peak for the 0.5Ni-Si/MgO indicating the high separation of photo-excited electrons, which was likely to be resulted from the hierarchically ultrathin nanosheets of catalyst.

**Figure 8:**
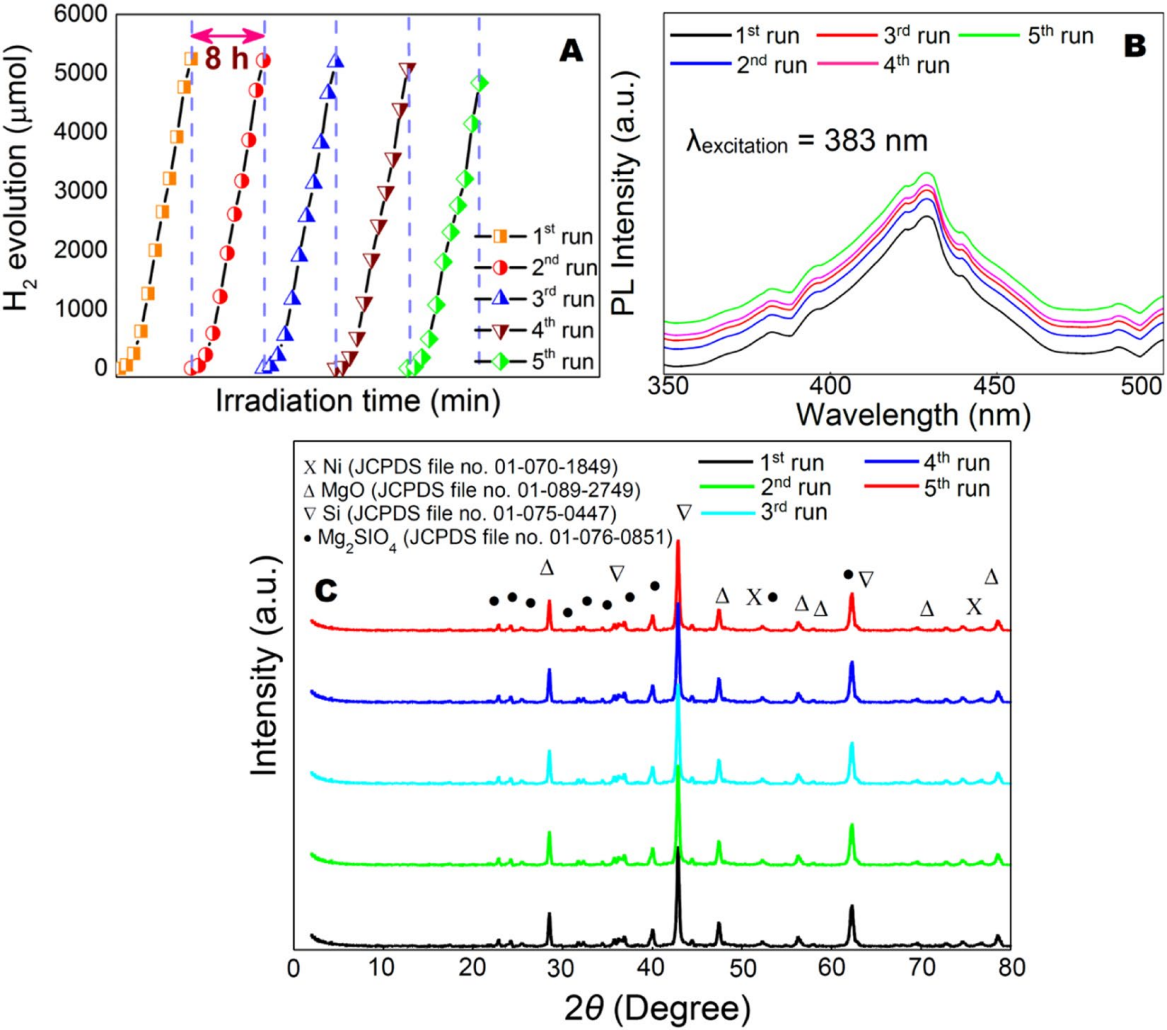
(**A**) Cycling test of photocatalytic stability of the ultrathin 0.5Ni-Si/MgO nanosheets. (**B**) PL spectra of the ultrathin 0.5Ni-Si/MgO nanosheets after five consecutive recycling test. (**C**) XRD pattern of 0.5Ni-Si/MgO for successive reuse of 5^th^ cycle.

**Figure 9:**
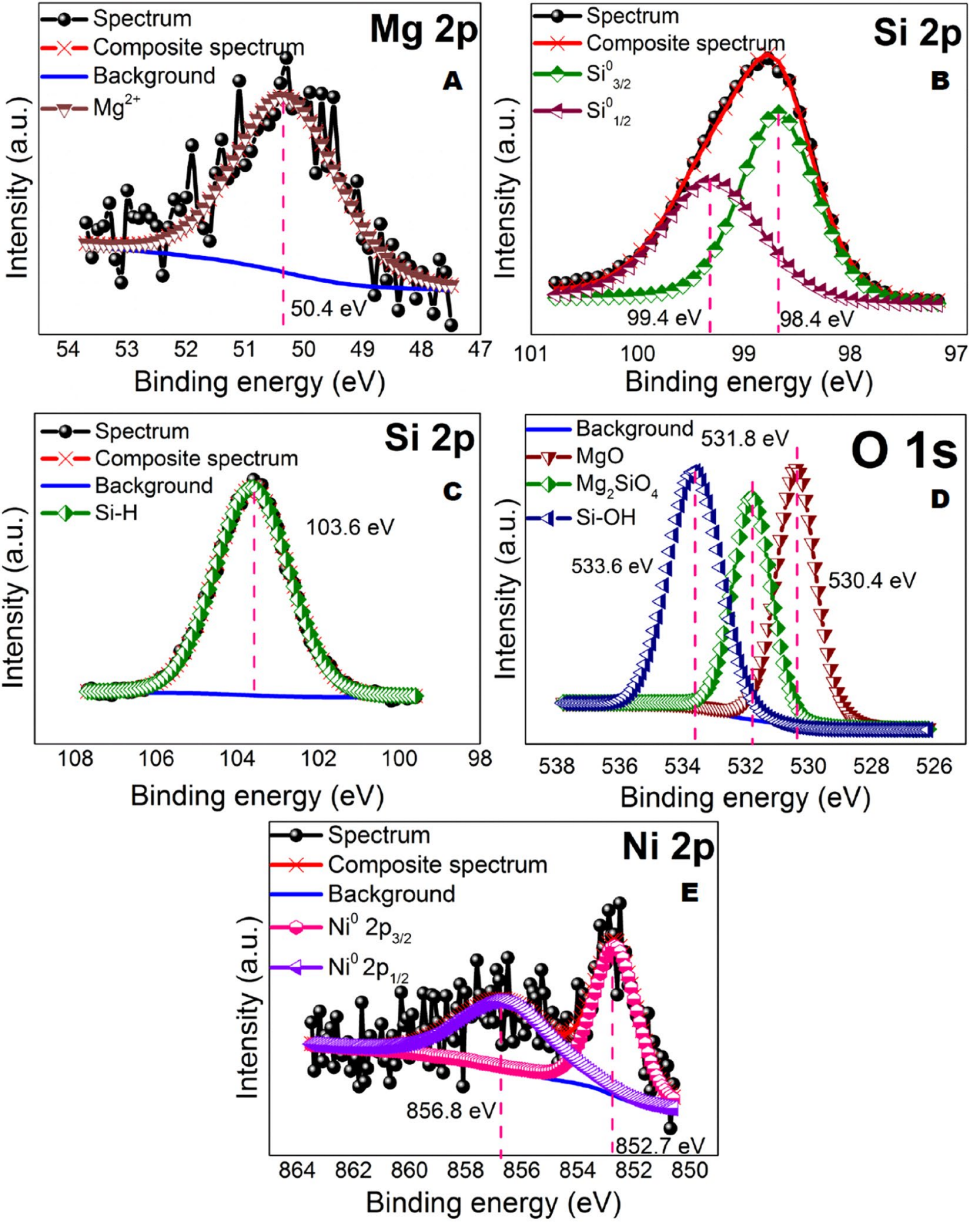
Chemical characterization of reused 0.5Ni-Si/MgO catalyst after 5^th^ cycle. (**A**) XPS spectra of Mg 2p (**B**,**C**) XPS spectra of Si 2p. (**D**) XPS spectra of O 1 S. and (**E**) XPS spectra of Ni 2p for 0.5Ni-Si/MgO catalyst.

Stability of the phocatalyst was carried out by recycling test. No noticeable loss of hydrogen production after progressively reuse was observed under the visible light irradiation (Fig. 8A). Through the reaction condition for the different research experiments was not same, the ultra-thin nanosheet photocatalyst observed in this experiment displayed relatively higher compared with the noble-metal-free photocatalyst^18,36,37,47^. Such high performance of hydrogen production from noble metal free photocatalyst has not been reported to the best of our knowledge. The lower decaying rate of PL signals as evident from Fig. 8B for ultrathin nanosheets, greatly retain their photocatalytic activity stable^65^. This noble metal-free efficient photocatalyst with remarkable photostability is highly desirable for industrial application. To gain further insight into the difference either in the morphology or in the chemical structure of catalyst after 5^th^ cycle, characterizations of the used catalyst were performed by using XRD, XPS, FESEM, HRTEM. No observable difference in the chemical structure of photocatalyst after successive reuse was identified by XRD (Fig. 8C) and XPS (Fig. 9A–D). However, the presence of additional peak is confirmed by fitting the high energy shoulders on the metallic line at energy of about 857.5 eV (Fig. 9E). The peak evidenced by XPS studies can be assigned to the formation of Ni(OH)_2_ in the catalyst after 5^th^ consecutive cycles^66^. It is important to point out that XPS only detects at the top 5–10 atomic layers of the sample^67^. However, Ni(OH)_2_ phase was not detected by XRD analysis in the sample after the 5^th^ consecutive cycles (Fig. 8C). Behr-Andres *et al*.^68^ reported that the XRD cannot detect any phase if the phase is either amorphous in nature or not enough volume to produce peaks of considerable intensity in XRD. A related observation has been reached by Chen *et al*.^69^ that the phase contents either less than 5% or extremely thin cannot detect in XRD analysis. In addition, the incorporation of small crystallites with randomly oriented phase of the sample into the bulk lattice might not contribute to show XRD peaks, as suggested by Köferstein *et al*.^70^. The results demonstrated that the Ni(OH)_2_ phase identified by XPS might not detect by XRD due to it’s presence of either amorphous or extremely thin phase in the reused sample.

**Figure 10:**
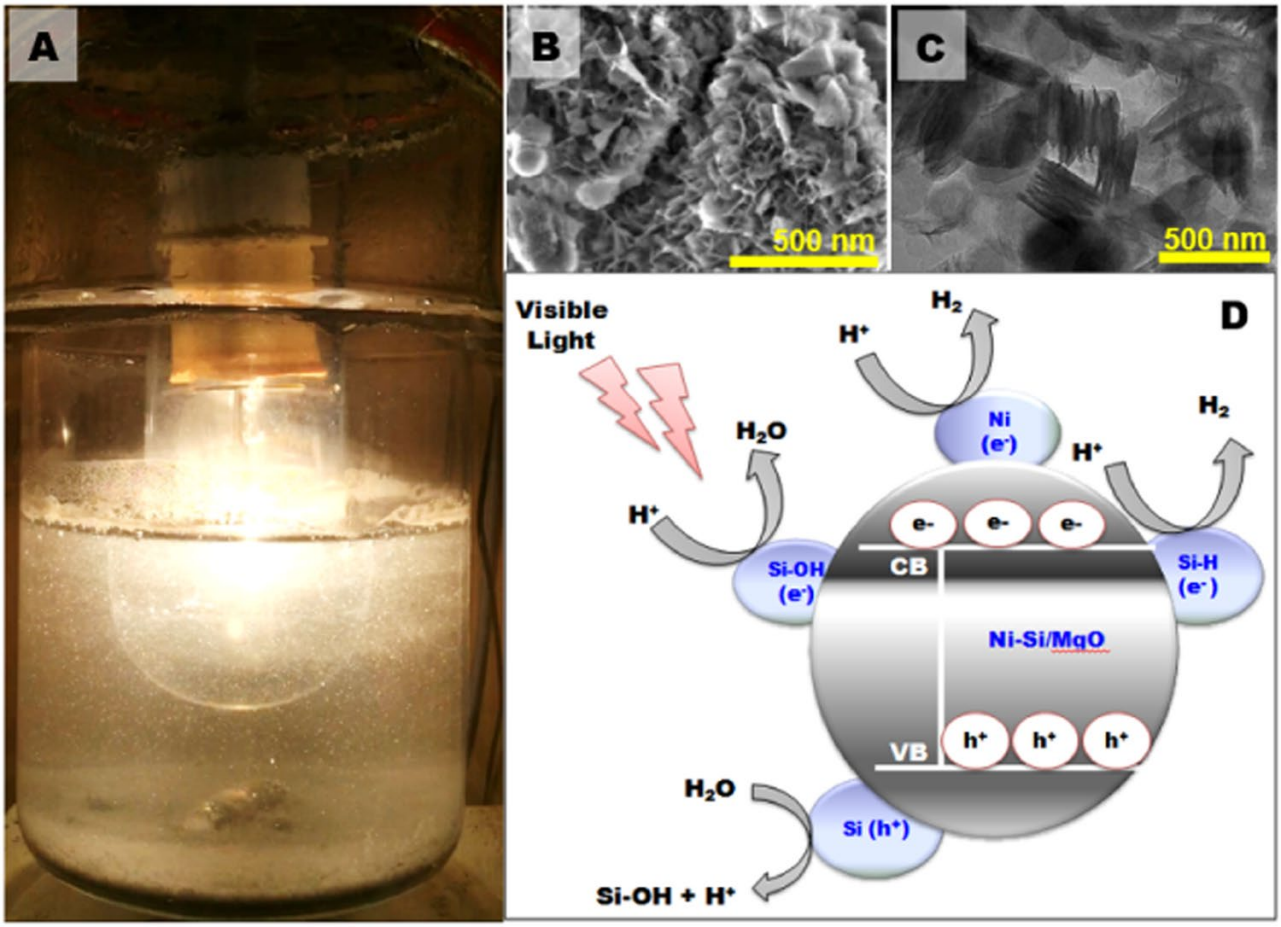
(**A**) Photography of photocatalytic reaction performed in a vessel under visible light illumination. (**B**) SEM image of the used 0.5Ni-Si/MgO photocatalyst. Inset: magnified image of used 0.5Ni-Si/MgO catalyst. (**C**) Transmission electron micrograph of used 0.5Ni-Si/MgO catalyst after 5^th^ cycle. (**D**) Proposed photocatalytic mechanism of Ni-Si/MgO photocatalyst under visible light irritation.

The photography of hydrogen evolution in photocatalytic vessel is shown in Fig. 10A. A significant difference in the morphology of used catalyst was observed (Fig. 10B,C). After few cycle of successive reuse, stacking of ultrathin nanosheets structured morphology were broken into some small nanoplates, producing novel flower-like hierarchically nanostructures assembled by large number of nanosheets with high uniformity (Fig. 10B). The intersection of nanosheets structures produced flower-like porous structure, as evident from TEM (Fig. 10C). The internal field at the interface of nanosheets facilitates the spatial separation of the photogenerated carriers, as widely reported in the literature^71^. Based on the results and discussion above, a probable mechanism for the improved photocatalytic activity of hierarchically ultrathin Ni-Si/MgO nanosheets can be proposed (Fig. 10D). It is usually known that absorption of solar light, photoexcitation, departure of electrons are main stages of photo-irradiated reaction. The excited electrons transmit to the surface species (Si-OH and Si-H) of conduction band and leaving behind the holes in the valance band. Part of these electrons would access to the conduction band of Ni and these electrons participate immediately in the photocatalytic reaction, leading to hole-electron separation. As previously reported^72^ H_2_ evolution could be facilitated by the nickel supported photocatalyst. Hence, the incorporation of Ni particle in the interlayer of catalyst may serve as reduction sites for the formation of H_2_. An opposite flow of holes (h + ) from the valence band to the Si-h + is expected. Recombination of major photoexcited electrons and holes and participation only a portion of electrons in the photocatalytic reaction causes in lower activity. The photogenerated electrons and holes from porous ultrathin layer of catalyst can be spatially distributed at the more active sites of co-catalysts (Ni, Si-OH and Si-H species) which promote to the accumulation of more electrons and holes on porous nanosheets. It has been shown by the several researchers^47,73^ that low possibilities of oxygen generation on Si could be associated with the surface bonds of Si-H and Si-OH on Si catalyst. These results provide substantial arguments to explain the mechanisms that occur at Si surface for the photoassisted water splitting. Optimizing and stabilizing the surface functions of catalyst could be a further step of research to improve the catalyst efficiency. Finally, we anticipate that the work presented here may open up new insights for the utilization of hierarchically porous ultrathin Ni-Si/MgO nanosheets photocatalyst as an alternative to noble metals supported catalyst for effective photocatalytic hydrogen production.

## Conclusions

In summary, a visible-light-driven Ni-Si/MgO catalyst system has been developed using a straightforward method based on a solid phase reaction strategy to make nanostructured ultrathin catalyst for efficient H_2_ evolution without adjusting pH, applied voltage, sacrificial agent or electron donor. It could be mentioned that the surface properties of photocatalyst is the one of the influencing factor to increase the H_2_ evolution for Ni-Si/MgO catalyst system. The demonstration of maximum hydrogen evolution from this precious-metal-free system is 714 µmol h^−1^, which can be compared to the previously reported for any noble-metal-free water-splitting photocatalyst. Exhibiting exceptional activity and the long-term stability of photocatalyst thus promote for practical implementation. Considering the ultrathin nanosheet morphology and high photocatalytic activity of Ni-Si/MgO catalyst, we believe that combining the other semiconducting materials with smaller band gaps will enable further improvements of photocatalytic H_2_ evolution. More prominently, the facile fabrication process for the nanosheet assembly of photocatalyst developed here may be a key advancement for the improvement of solar light driven clean fuel conversion technologies.

### Experimental sections

#### Synthesis of Ni and Si precursor

Semiconductor based photocatalyst was fabricated using solid combustion method in a self-customized reactor. High crystallite silica powder at nanoscale was obtained by wet acid and thermal treatments. Firstly, the rice husk (collected from sekinjang, Malaysia) was purified using 20% hydrochloric acid (pH = 2) at 90 °C for 1 h. The collected samples were washed with water and dried in an oven for 4 h. Calcination of the sample was performed at 800 °C in an air for 3 h under ramping 5 °C min^−1^. Silica powder was collected after calcination steps. Sodium borohydride and nickel salts were used to prepare Ni_*x*_B catalyst. Typically, 5 wt%NaBH_4_ (98%, Fisher Scientific) solution cooled in ice water bath (5–7 ^o^C) and adjusted pH 12 of the solution under stirring. The 6 wt%NiCl_2_ (99%, Fisher Scientific) solution added slowly to the NaBH_4_ (Fisher Scientific) solution slowly for 3 h to avoid violent hydrogen evolution. The filtration of black color precipitates was performed and washed with water to remove remaining chloride ions. Finally the sample was dried in an oven at 150 °C.

### Synthesis of photocatalyst

Nickel-Silicon/magnesium oxide photocatalyst was prepared by one-pot facile magnetothermic reduction process in a closed reactor. In a typical procedure, grounding of 1 g silica powder with different amount of NiB ranging from (0.5–0.7 g) and 1.1 mg of Mg powder (Sigma-Aldrich Co, LLC., nanopowder). The heat generated by exothermic reaction was avoided by spreading the catalyst at the bottom of the reactor. Tube furnace was used to heat the reactor (carbolite G, model 12/1200) to 750 °C for 3 h with 5 °C min^−1^ increasing rate. The reactor was collected from the furnace after solid phase reaction at temperature below 100 °C. The resultant catalyst was used in water for reaction without adjusting pH, applied voltage, sacrificial agent or electron donor.

### Measurement of H_2_ evolution

**Figure 11:**
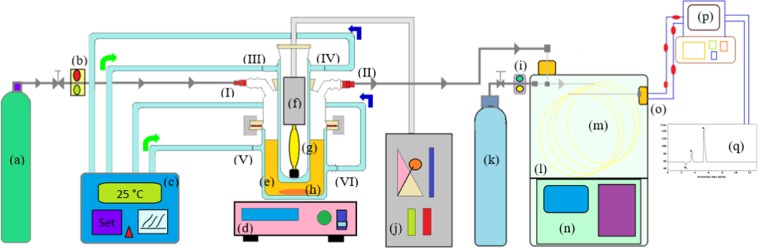
A schematic of photochemical water splitting reaction for hydrogen production. Argon gas (**a**), flow controller (10 ml min^−1^) (**b**), thermostatic bath (**c**), magnetic stirrer (**d**), reactor vessel (**e**), light source (**f**), lamp jacket (**g**), magnetic bar (**h**), flow controller (20 ml min^−1^) (**i**), power generator (**j**), nitrogen gas (**k**), column oven (**l**), column (**m**), oven controller system (**n**), thermal conductivity detector (**o**), data acquisition system (**p**), recorder (**q**), air inlet (I), air outlet/ sample gate (II), cooling water inlet (III,V), cooling water outlet (IV,VI).

Photocatalytic reaction was performed without using any sacrificial reagent (Fig. 11) equipped with a vessel for reaction, vacuum line and a port for gas sampling connected directly to an online gas chromatography (GC, Agilent technologies 7890 A). Thermal conductivity detector and molecular sieve (Part number 19095P-MSO, Agilent US) were connected with the GC. Argon gas was used as a carrier gas.

In a typical run, 1.0 g of resultant Ni-Si/MgO catalyst was dispersed in pure water (380 mL) without adjusting pH, applied voltage, sacrificial agent or any electron donor. The dead volume of the pyrex reactor used for reaction was 500 mL. A tape water bath was applied to keep the reaction vessel cool. Evacuation process was applied for degassing the reaction system before irradiation. The light irradiation was a 450 W xenon lamp (l > 400 nm) through a quartz cooling jacket. Two μl of sample was injected with carrier gas N_2_. The comparison was made with standard retention time of H_2_ to calculate generation quantity of H_2_. To calculate the H_2_ yield, a calibration curve was generated by injecting known amount of H_2_ and integrating signal intensities. The measurement details were given in Table S1. To evaluate the cycling test, photocatalytic hydrogen evolution of the ultrathin 0.5Ni-Si/MgO nanosheets were performed under visible light (>400 nm) for five cycles. The H_2_ production rate (Eq. 4) was calculated from the ratio between the moles of H_2_ generated in the reaction and the duration of reaction time.

4 $${\rm{Hydrogen}}\,{\rm{production}}\,{\rm{rate}}=\frac{{\rm{Hydrogen}}\,{\rm{generation}}}{{\rm{Reaction}}\,{\rm{time}}}$$

### Sample characterization

Detection of crystal structure of the sample was performed XRD (Model: Shimadzu, XRD6000 with a PIXcel 1D detector with Cu K*α* radiation). The current 30 mA and operating voltage 2.7 kV were maintained. A range of 2*θ* value was scanned from 10–80° with scan rate of 2° min^−1^. Peak elucidation was performed by matching the JCPDS library.

UV-Vis-NIR absorption spectra (UV-vis) were obtained by the diffuse reflection method using a Perkin spectrometer and BaSO_4_ as a reflectance standard. Conversion of absorbance was performed using reduced reflectance technique. The surface structure of the catalyst was measured by FESEM (Model SU8000). The voltage was maintained 12 kV and distance for measuring maintained ~4 mm. To determine the chemical composition existence, EDX was performed on ThermoscientificNoran System 7 with Nanotrace detector.

HRTEM (HT-7700) was used to observe the structure of photocatalyst. The point resolution and accelerating voltage were used 0.09 nm and 200 kV respectively under high vacuum mode. The oxidation states of catalyst were examines by XPS (Quantera II) radiated 1486 eV using Al Kα. A monochromator X-ray light source was used operating at 25.6 W. The system was calibrated by the 284.8 eV of the binding energy for carbon. The measurement of electron-hole recombination property was carried out by photoluminescence (PL, Elmer LS55, xenon (Xe) lamp with 383 nm wavelength).

## Supplementary information

Supplementary information.
