# Supplementary material for: Ultrathin Assembles of Porous Array for Enhanced H2 Evolution
Source: Sci Rep. 2020 Feb 11;10:2324. doi: 10.1038/s41598-020-59325-4 (PMC7012925; doi:10.1038/s41598-020-59325-4)
Supplement: Supplementary file 1 — Supplementary information. [file 41598_2020_59325_MOESM1_ESM.pdf]

Supporting Information

Ultrathin assembles of porous array for enhanced H_2 evolution

Aminul Islam[2*], Siow Hwa Teo[1,3], Md. Rabiul Awual[4], Yun Hin Taufiq-Yap[1,3*]

[1]Chancellery Office, Universiti Malaysia Sabah, 88400 Kota Kinabalu, Sabah, Malaysia.

[2]Department of Petroleum and Mining Engineering, Jashore University of Science and Technology, Jashore-7408, Bangladesh.

[3]Catalysis Science and Technology Research Centre, Faculty of Science, Universiti Putra Malaysia, 43400 UPM Serdang, Selangor, Malaysia.

[4]Materials Science and Research Center, Japan Atomic Energy Agency (JAEA), Hyogo 679–5148, Japan.

*corresponding and requests for materials should be addressed to A.I. (email: aminul_pme@just.edu.bd) or Y.H.T.Y. (email: taufiq@upm.edu.my)

Table S1

Injection	Volume of injection (mL)	(L)	GC area	Concentration (%)	Molar (mol L^{-1})	Moles of H_2 in 0.25 ml mol
1	0.25	2.5×10^{-4}	738.55	5	0.00223	5.58×10^{-7}
2	0.25	2.5×10^{-4}	738.55	5	0.00223	5.58×10^{-7}
3	0.25	2.5×10^{-4}	738.55	5	0.00223	5.58×10^{-7}

Water splitting: GC injections

Injection	GC area	Time (min)	Mole in 0.25 mL	Mole in 30 cm^3	H_2 Evolution (μmol)
140	289.603	417	2.18641E-07	2.62369E-05	4957.237
141	271.813	420	2.0521E-07	2.46252E-05	4981.862
142	269.252	423	2.03277E-07	2.43932E-05	5006.256
143	284.089	426	2.14478E-07	2.57374E-05	5031.993
144	299.492	429	2.26107E-07	2.71328E-05	5059.126
145	296.261	432	2.23667E-07	2.68401E-05	5085.966
146	290.797	435	2.19542E-07	2.63451E-05	5112.311
147	289.569	438	2.18615E-07	2.62338E-05	5138.545
148	287.329	441	2.16924E-07	2.60309E-05	5164.576
149	286.363	444	2.16195E-07	2.59434E-05	5190.519
150	285.772	447	2.15749E-07	2.58898E-05	5216.409
151	284.765	450	2.14988E-07	2.57986E-05	5242.207
152	282.881	453	2.13566E-07	2.56279E-05	5267.835
153	280.618	456	2.11857E-07	2.54229E-05	5293.258
154	278.887	459	2.10551E-07	2.52661E-05	5318.524
155	275.991	462	2.08364E-07	2.50037E-05	5343.528
156	274.918	465	2.07554E-07	2.49065E-05	5368.435
157	272.119	468	2.05441E-07	2.46529E-05	5393.088
158	270.776	471	2.04427E-07	2.45313E-05	5417.619
159	268.654	474	2.02825E-07	2.4339E-05	5441.958
160	268.654	477	2.02825E-07	2.4339E-05	5466.297
161	285.665	480	2.15668E-07	2.58801E-05	5492.177

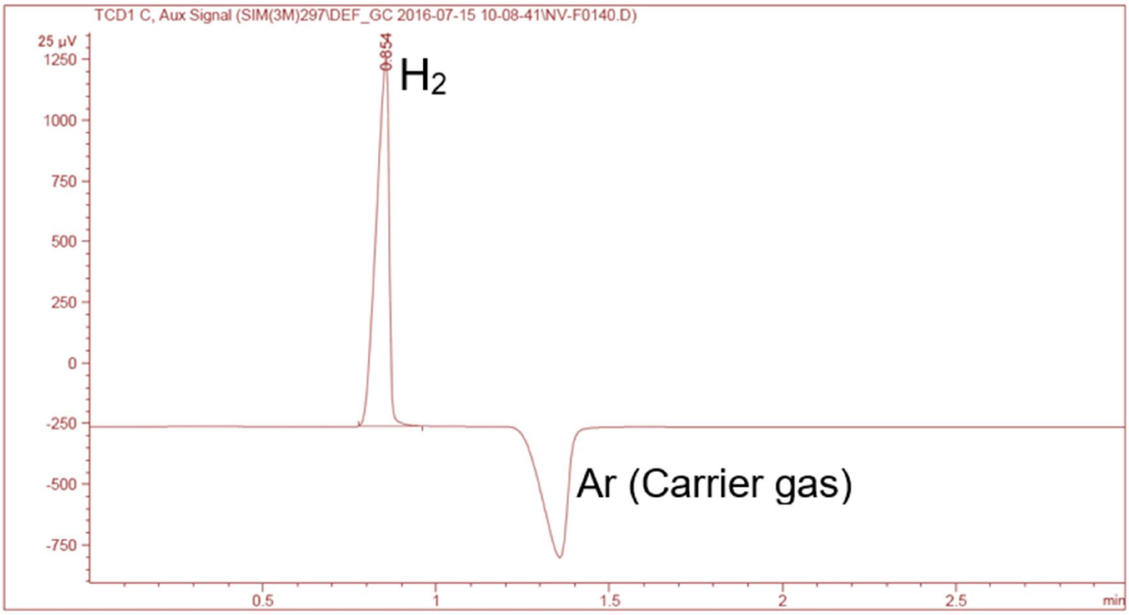

Supplementary Figure S1. A typical GC trace of evolved Hydrogen.

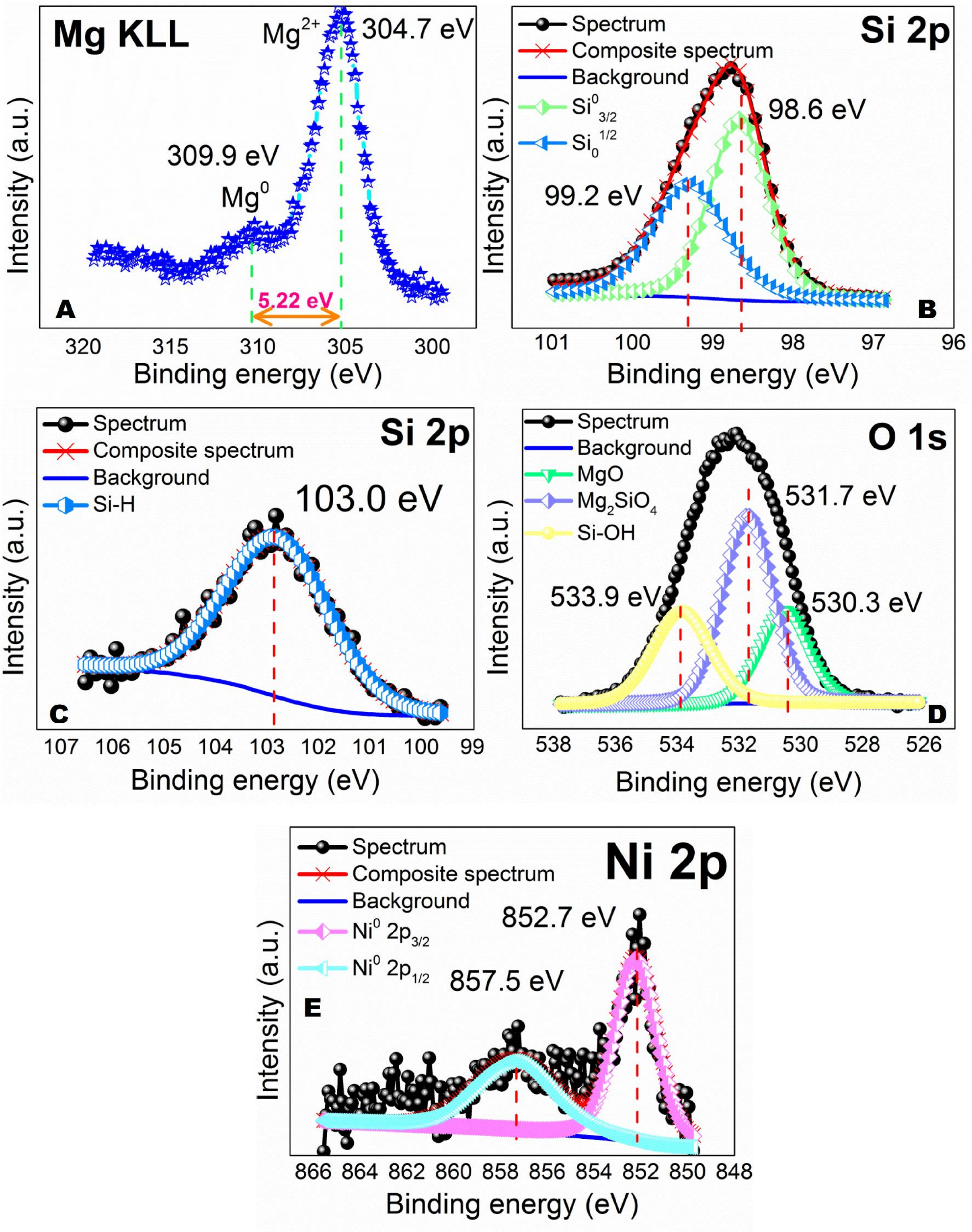

Supplementary Figure S2. Chemical characterization of 0.7Ni-Si/MgO catalyst. (A) XPS spectra of Mg KLL (B-C) XPS spectra of Si 2p. (D) XPS spectra of O 1S. and (E) XPS spectra of Ni 2p for 0.7Ni-Si/MgO catalyst.
